# UK Postal Delivery Workers’ Occupational Sun Safety: Using Behavior Change Theories to Identify Intervention Pathways

**DOI:** 10.3390/ijerph16193712

**Published:** 2019-10-02

**Authors:** Jonathan Houdmont, Raymond Randall, Alistair Cheyne, Shaun Davis, Hannah Evans, Joanne Faichney

**Affiliations:** 1 Centre for Organizational Health and Development, School of Medicine, University of Nottingham, B Floor, Yang Fujia Building, Jubilee Campus, Wollaton Road, Nottingham NG8 1BB, UK; msxjf9@nottingham.ac.uk; 2 Management School, University of Sheffield and Loughborough University, Sheffield, S10 1FL, UK; r.randall@sheffield.ac.uk (R.R.); hannah.evans@sheffield.ac.uk (H.E.); 3 ESSCA School of Management and School of Business and Economics, Loughborough University, Loughborough, LE11 3TU, UK; A.J.T.Cheyne@lboro.ac.uk; 4 Royal Mail Group, Slough, SL3 8AQ, UK; shaun.davis@royalmail.com

**Keywords:** postal delivery workers, Theory of Planned Behavior, Transtheoretical Model, stage of change, sun safety, solar radiation

## Abstract

Postal delivery workers have substantial sun exposure. In the United Kingdom (UK) a high proportion of workers possesses a sun sensitive skin type. This population is at elevated risk for skin cancer, yet uptake of sun safety practices is low. Studies are needed to identify the underlying factors that contribute to the uptake of occupational sun safety practices that may be targeted during behavior change interventions. This study integrated the Theory of Planned Behavior (TPB) and the Transtheoretical Model’s stages of change (SoC) as guiding frameworks to identify underlying beliefs that influence UK postal delivery workers’ uptake of occupational sun safety practices. Thirty-four workers participated in semi-structured interviews that used the SoC to establish current receptiveness to and adoption of two sun safety practices (using sunscreen of at least sun protection factor (SPF) 30 on exposed skin and wearing a wide-brimmed hat when working outdoors in the summer). Beliefs underlying current practices were elicited in accordance with the TPB and stratified by the SoC. For sunscreen use and wearing a wide-brimmed hat, 64% and 3% of participants were in the action or maintenance SoC, respectively. Behavioral and control beliefs differed by SoC, with those in the earlier stages more likely than those in the latter stages to report negative attitudes to, and difficulty enacting, sun safety practices. Normative beliefs concerning the views of colleagues and employers towards sunscreen were relatively consistent across the SoC. This study highlights the need for tailored and targeted behavior change interventions. The SoC-stratified accounts of the influence of TPB components on behavior provide a basis for bespoke interventions that reflect inter-individual and inter-practice differences in their working mechanisms.

## 1. Background

Solar ultraviolet (UV) radiation is the primary risk factor for all forms of skin cancer [1]. In Britain, excess exposure to solar UV radiation accounted for an estimated 90% of cases of malignant melanoma in males and 82% in females in 2010 [2], with registration rates for melanoma skin cancer showing a 56% increase for males in England in the period 2002–2011 [3]. Occupational solar UV radiation exposure is estimated to account for 3.2% of cases of cutaneous malignant melanoma in males and 0.9% in females in Britain, amounting to 48 deaths (2012) and 241 new registrations (2011) per annum [4]. The World Health Organization’s Global Solar UV Index (UVI) describes the level of solar UV radiation at the earth’s surface and facilitates comparisons across locations [5]. Index values are grouped into exposure categories, with scores of <3 indicating low exposure, 3–5 moderate exposure, 6–7 high exposure, 8–10 very high exposure, and ≥11 extreme exposure. Sun protection is recommended at a score of ≥3. The UK government’s online UV radiation data mapping tool (https://uk-air.defra.gov.uk/data/uv-data) provides official UVI data gathered from the Reading University monitoring site (located in the south of England). These data show a mean maximum daily index score for 2017 of 2.99. During the official 94-day summer period (21st June to 22nd September 2017) the mean maximum daily score rose to 4.85, during which time it ranged from 1.10 to 8.00, with low exposure on 11.7% of days, moderate exposure on 56.9% of days, high exposure on 27.7% of days, and reaching the very high exposure threshold on a single day.

Postal delivery workers are an important population to target for skin cancer prevention efforts. Royal Mail Group (RMG) is the United Kingdom’s (UK) designated universal postal service provider, employing 88,000 postal delivery workers in 2018. These employees work outdoors for an average of 5.4 h per day and two-fifths report their skin type as “pale” or “very pale” [6]. These are factors that place them at increased risk for solar UV radiation exposure and skin cancer [7]. Occupational exposure to solar UV radiation is a modifiable risk factor. As such, the purpose of the current study is to apply empirically established behavior change theories to identify factors underlying the uptake of two sun safety practices among UK postal delivery workers. This knowledge is needed to inform the content and targeting of occupation-specific interventions.

### 1.1. Postal Delivery Worker Sun Safety Research 

Most postal delivery worker sun safety research has been conducted in geographical locations that experience prolonged periods of intense sunshine [8] and has often involved a high proportion of participants from ethnic groups at low risk for sunburn. For instance, research involving United States Postal Service employees has centered on Southern California [9], with Project Sunwise, a large-scale study involving more than two thousand Southern Californian letter carriers, finding that 49% identified as non-white with only 5% of respondents reporting high sun sensitivity, e.g., always burn, unable to tan in response to first exposure to summer sun [10,11,12]. Project Sunwise remains the sole postal delivery worker sun safety intervention study and focused on wearing a wide-brimmed hat and using sunscreen: intervention group participants were twice as likely as those in a control group to regularly use sunscreen at two-year follow up [13].

Contrasting levels of sun susceptibility among postal delivery workers allied with stark differences in the frequency and the intensity of sunshine within the research context suggest that existing intervention research findings may not transfer well to temperate climate zones (fewer hours and less intensity of sunshine) and where high proportions of postal delivery workers are of a high-risk skin type. Research is needed that is specific to temperate climate zones and can be used to develop and evaluate context-specific occupational sun safety interventions.

There is a small body of recent sun safety research involving postal delivery workers in temperate climate zones. In New Zealand, 41% of postal delivery workers reported that their skin always or usually burns in response to the sun, with sun safety knowledge lower among postal workers than in other outdoor worker groups despite high provision of sun safety protective and precautionary measures in the postal sector [14]. An earlier study in New Zealand found that postal delivery workers were more likely to report workplace support for sun safety than workers from other industrial groups [15]. Yet, whereas the former study found that sun safety knowledge was poor among postal delivery workers, the latter found that postal delivery workers were more likely than workers from other outdoor occupational groups to use sunscreen. A German study involving a small sample of 20 postal service workers found that all participants worked outdoors for at least four hours per day, with 70% indicating they received no employer-provided sun protection measures [16].

In a survey on sun safety knowledge and practice involving 1153 RMG employees [6], ≥50% of respondents reported correct knowledge on three domains: the need to wear sunscreen on a cloudy day, the need to wear sunglasses to protect the eyes from solar UV radiation, and that sun exposure is a key risk factor for skin cancer. However, fewer than half of respondents reported correct knowledge in relation to neck protection, the need for repeated administration of sunscreen throughout the day, and the need for sun protection when working outdoors for less than one hour. Mixed results were similarly observed for sun safety practices, with the majority of respondents reporting good practice on four domains (using a shade/cover when working in the sun, wearing long-sleeved loose-fitting tops and trousers, using sunscreen, checking the UV index) but as few as one-fifth reporting wearing sunglasses and drinking plenty of water when working outdoors in the summer.

These findings provide a useful initial evidence base concerning postal workers’ occupational sun safety practices within temperate climate zones. The research has identified effective practices that are not sufficiently widespread and warrant being further encouraged. However, the research provides little by way of instruction as to how positive behavior change might be achieved. Effective intervention requires understanding of the underlying factors that contribute to low uptake of sun safety practices; knowledge in this regard could enable these underlying factors to be taken into account in the design and targeting of interventions. 

### 1.2. Behaviour Change Theory and Sun Safety Research

The aim of this study was to identify pathways for intervention through analysis of the factors influencing postal workers’ current sun safety practices. Adoption of sun safety measures involves active choices to make cognitive and behavioral adjustments. Contemporary psychological theories of behavior change can be used to identify potential pathways through which interventions lead to and maintain these adjustments. These theories provide a guide for the probing of potentially relevant influences, offer a structure for the reporting of findings, and can inform the focus of interventions to promote desirable behavior. The Theory of Planned Behavior (TPB) [17] has provided a framework for a wealth of behavior change research and provides clear guidance for the identification of the underlying beliefs determining behavior that may subsequently be targeted in behavior change interventions. In TPB, the proximal determinant of actual behavior is intention to behave. This intention is determined by three principal components: (i) attitude towards the behavior, i.e., positive or negative evaluations of performing the behavior (behavioral beliefs), (ii) subjective norms towards the behavior, i.e., perceived social pressure to perform or not perform the behavior (normative beliefs), and (iii) perceived behavioral control, i.e., control over the ability to perform or not perform the behavior (control beliefs).

The TPB has been used to examine predictors of sun safety practices. For instance, Australian adults’ behavioral beliefs concerning ability to tan, normative beliefs about friends’ views on sun protection, and control beliefs about forgetfulness, inconvenience, duration of sun exposure, and fashion status of sun-protective clothing were found to be significantly linked to sun-protective behavior [18]. Another Australian study demonstrated that the model’s three antecedent components predicted mothers’ behavior in respect to sun protection for their children [19]. A US study found that TPB components accurately predicted students’ salon tanning intentions, with attitudes towards tanning being the strongest predictor followed by subjective norms (the extent to which people who were important to them thought they should use a tanning salon) and perceived behavioral control (ease of using a tanning salon) [20]. In qualitative research the TPB was used to gain insights into facilitators and barriers of Australian adolescents’ sun protection behaviors, with attitudes towards tanning (behavioral beliefs) identified as a key predictor alongside parental reminders and context, i.e., planned versus spontaneous events (normative beliefs) and perceived control over ability to apply sunscreen (control beliefs) [21]. The TPB was further used in a study of UK construction workers to shape questions on beliefs concerning the advantages and the disadvantages of performing sun safety behaviors, the most important individuals or groups that would approve or disapprove of performing these behaviors, and barriers and facilitators to performance of the desirable behaviors [22]. That study also used the TPB to quantitatively examine the extent to which the beliefs accounted for variance in sun safety behavioral intentions pre- and post-intervention. The TPB provides a strong theoretical basis for the development of sun safety intervention content and targeting. Therefore, we used it as a guiding framework for the exploration of postal delivery workers’ accounts of the factors underlying occupational sun safety practices.

### 1.3. Readiness for Behavior Change and Intervention Tailoring 

The preceding review demonstrates that the TPB provides a basis for the development of interventions centered on behavioral beliefs, normative beliefs, and control beliefs that are specific to the target population. Health promotion interventions are particularly effective when messages are tailored according to participants’ stage of readiness to change their behavior [23]. Implicit within this Stage of Change (SoC) perspective is the notion that TPB beliefs may exhibit stage-specificity and by extension different types of TPB-based health promotion communications are needed for individuals residing at each stage. Prochaska and DiClemente’s Transtheoretical Model (TTM) [24] provides a useful framework to understand readiness to change sun safety behaviors [22,25,26,27,28,29]. According to the model, individuals in the process of eliminating health-compromising behaviors or acquiring desirable behaviors pass through five specific stages. In the first stage, precontemplation, individuals have no intention to change the behavior or are resistant to consider modifying the behavior. Those in the second stage, contemplation, have thought about change but are not yet ready to act. In the third stage, preparation, a decision has been made to change behavior and plans may have been put in place to enact this. Individuals in the fourth stage, action, have changed their behavior, usually recently (many studies apply a window of the last six months). The fifth stage, maintenance, is characterized by a sustained change in behavior, generally of at least six months. Some studies include a final sixth stage, termination, where individuals *“display total mastery over the target behavior, experiencing no temptation to revert to old habits”* (p. 182) [30]. A relapse stage may also be included to recognize that change does not always proceed sequentially. 

TTM sun safety studies have consistently demonstrated that beliefs exhibit some stage specificity. For example, among a non-occupational sample of Australian adults [30] it was found that those in the precontemplation stage were significantly less concerned about the possibility of contracting skin cancer than those in the contemplation and the action stages. These participants also demonstrated an optimism bias, believing that their chances of contracting skin cancer were lower than the chances reported by those in the later stages. Findings such as these suggest that to be maximally effective sun safety interventions should be tailored to reflect these variations in stage-specific beliefs. The empirical evidence supports this notion, with sun safety interventions tailored to SoC more effective than generic interventions that fail to take account of barriers and facilitators to change dominant at each stage [31]. There remains, however, a lack of research integrating the TTM and the TPB to facilitate the design of such interventions. 

### 1.4. Aims of the Study

In this study, we applied the TPB to identify and analyze the factors that UK postal delivery workers report as influencing their current usage of two sun safety practices classified according to SoC. The results can be used to inform the content and targeting of behavior change interventions that are based on established behavior change theory and reflect SoC diversity of the target population.

## 2. Method

### 2.1. Participants and Procedure

RMG regional health and safety representatives invited depot managers within their jurisdiction to participate. Regions were selected on the basis of being within one-hour of travel for the researchers (J.H., R.R., J.F.) and encompassed the midlands, the north of England, and Northern Ireland. Depot managers that agreed to take part were asked to provide a quiet office for interviews and a list of postal delivery workers willing to be interviewed on an appointed day. All potential participants were provided with a detailed study information sheet. Written consent was obtained from those who chose to participate. Interviews were conducted between 07:00 and 09:00 before workers departed to commence their deliveries and took place during the summer months (June–September) of 2017 and 2018. Data were collected at this time because participants were likely to have been making frequent and recent choices about the adoption of sun safety measures and therefore able to accurately report on their current behaviors. Moreover, it was important to collect data at a time of year when the issue of sun safety was likely to be perceived as most relevant by prospective participants. Given that participation in the study was voluntary, with data collection conducted at a busy time during the work day, we wanted to maximize the perceived relevance of the study in order to encourage participation; though there exists potential for harmful solar UV exposure outside of this period, the topic is likely to be perceived as most relevant by outdoor workers during the summer months. Data collection ended only when thematic saturation had been achieved.

**Figure 1:**
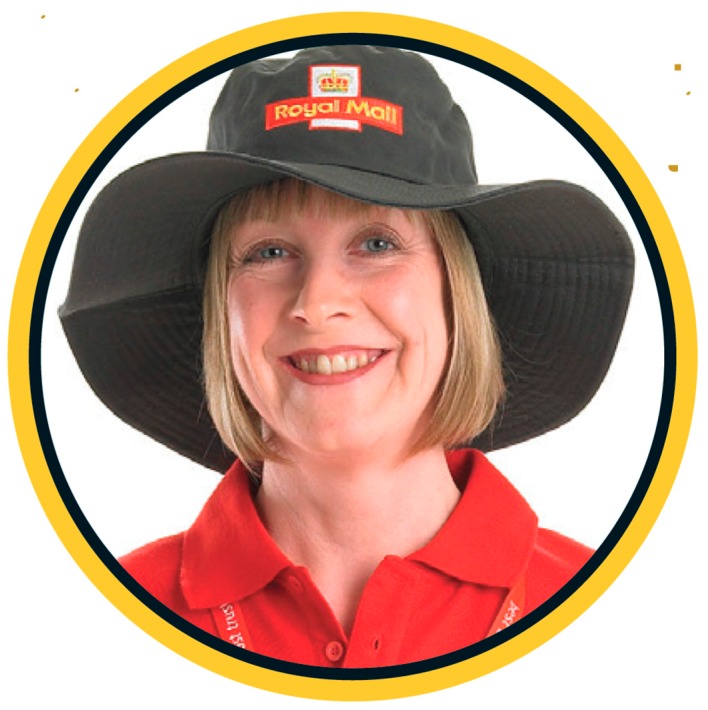
Royal Mail Group wide-brimmed hat.

A semi-structured interview schedule was used to elicit information on SoC and factors underlying current practice for two sun safety measures: using SPF30 (sun protection factor) or higher sunscreen on exposed skin and reapplying frequently as well as wearing a wide-brimmed hat when working outdoors in the summer months. These were selected on the basis that each has established effectiveness, is largely within the control of the individual worker, and has been shown to be a successful intervention target in previous US sun safety research involving postal delivery workers [13]. The RMG-issued wide-brimmed hat is shown in Figure 1. Employees may request a wide-brimmed hat at no charge from the RMG uniform website; RMG does not provide sunscreen. 

Current SoC for each sun safety measure was determined by a response of (i) “I do not do this and I am not thinking about starting” (precontemplation), (ii) “I do not do this but I am thinking about starting” (contemplation), (iii) “I do not do this but am planning to start in the next month” (preparation), (iv) “I do this but have only begun to do so this year/recently” (action), (v) “I do this and have done so for more than a year” (maintenance), and (vi) “I did this but no longer do so” (relapse). Participants were shown these descriptions of the stages and asked to indicate which best reflected their current practice. 

The response to the SoC question was followed by a set of questions to explore reasons for using or not using the measure. The questions were worded slightly differently according to whether the participant was in the precontemplation, contemplation, or relapse (inaction) stages or the preparation, action, or maintenance (action) stages. Based on the TPB principal components, example questions include: *“Do you think this is something that could be worth doing? Why?”* (behavioral beliefs); *“Is this something others expect you to do or think you should be doing?”* (normative beliefs), *“Is this something you are able to do? Why?”* (control beliefs).

Interviewees also supplied information on age and gender. Skin type was self-assessed using the SKINDEX poster that presents a color image and textual description of six broad skin types [32]. 

The mean interview length was 15 min (range, 11–21 min). Interviews were audio recorded using a digital voice recorder. In order to facilitate coding and analysis, recordings were transcribed verbatim and entered into NVivo for organizational support of the analysis. Ethical approval was obtained through the procedures of the Human Participants Sub-Committee of the Loughborough University Ethics Committee. 

### 2.2. Data Analysis 

To identify reasons underlying the uptake of sun safety practices, the dataset was analyzed using Braun and Clarke’s [33] six-stage method of thematic analysis: data familiarization, initial code generation, search for themes based on initial coding, review of themes, theme definition and labeling, and report writing. Initial coding of the full set of transcripts was undertaken by H.E., J.H. and R.R. each independently coded a sub-set of transcripts and identified largely identical codes to one another and HE. In the small number of cases where there was inconsistency discussion between HE, JH, and RR helped to clarify themes and reach consensus on codes and their definitions and labeling. The focus of analysis was on recurrent themes and sub-themes; in the development of themes, we used an inductive process with themes and descriptors iteratively generated to accommodate the data if existing themes were inadequate or better grouped within a higher-order theme. Higher-order themes were the elements of the TPB (behavioral beliefs, normative beliefs, control beliefs). Subsequently, themes were collapsed into a manageable number based on commonality of content. When similar reasons were mentioned by three or more participants, these were considered themes. The SoC within which each reason emerged was also recorded, resulting in the stratification of each theme of reasons by SoC. This allowed qualitative comparisons to be made between the beliefs mentioned by those at different stages and thus the identification of stage-specific and stage-generic themes. All authors reviewed and agreed upon the names of the themes within the final set. 

## 3. Results

A total of 34 interviews were conducted. The mean age of participants was 40.8 years (SD = 11.06), and all but three participants were male. The skin type distribution is shown in Table 1. 

The high proportion of participants with a sun sensitive skin type highlights the importance of sun safety research with this employee group. RMG does not hold data on employees’ skin type, preventing assessment of the extent to which this distribution of skin type is representative of the workforce in the regions involved in this research. However, this distribution is broadly consistent with that found in a large-scale survey of more than one thousand RMG employees [5]. The gender distribution in our study is consistent with that of the RMG workforce in the regions involved: 9% of our participants were female compared to 12% of the relevant RMG workforce.

### 3.1. Sunscreen

The stage distribution for wearing sunscreen of at least SPF30 on exposed skin and reapplying frequently when working outdoors in the summer is shown in Table 2. The results that follow therefore reflect differences between those within the early inaction stages and primarily those at the maintenance stage within the action stages. 

#### 3.1.1. Behavioral Beliefs 

Attitudes towards sunscreen centered on two themes—beliefs about the risk of skin cancer presented by both UK climate and personal characteristics, particularly skin type. Beliefs were largely stage-specific, with those in the earlier stages of change holding the view that the temperate UK climate does not present a risk of burning, precluding a need for sunscreen, *“We never get enough hot days and I’ve just never done it [used sunscreen] while working”* (contemplation); *“It would need over a couple of weeks at least before I would notice my skin darkening, by that stage it usually starts to rain again”* (precontemplation). In contrast, those in the action stages held that, although sun exposure is intermittent in the UK, the potential risk remains high, *“I always use it [sunscreen] because ultraviolet here in Britain’s just as strong as anywhere in Europe when the sun’s out”* (maintenance). Similarly, *“Over here you don’t think that you get burnt, but you do. Over here, you think we can just go out there in the sun. We’re not used to it, but I have started doing it because it has been really warm here and we are not used to it”* (action).

A distinction was evident across the stages of change for attitudes towards the need for sunscreen based on personal characteristics. Those in the inaction stages tended to perceive themselves being at low risk due to personal characteristics such as skin type, *“Even on holiday I rarely use sun cream, because I don’t burn. I just go brown, so you sort of don’t need to [use sunscreen]”* (precontemplation); *“I’m just fortunate enough to not really burn in that kind of weather, so I can get away with it”* (contemplation); and to a lesser extent, hair coverage, *“My arms are quite hairy so it takes me a long time to get a sun tan”* (precontemplation). 

Those in the action stages were more likely to acknowledge the higher risk associated with their skin type, often citing previous experiences of sunburn as a motivating factor, *“I always plaster myself in it. I burn easily and have learned the hard way”* (maintenance); *“I get quite badly burned on the face so I use factor 50 on my face no matter what”* (maintenance), with some noting the implications of sun burn for work ability, *“After a burn it’s hard to walk, you don’t want to work, you are dehydrated, it’s just something you don’t want to go through again”* (maintenance). 

It was not uncommon for those in the action stages to indicate that sunscreen use was motivated by personal or family and friends’ experiences of skin cancer, *“The reason I do it is that my mum had skin cancer on her face, so that is something I’m mindful of. Although I wouldn’t necessarily burn in the sun here, she wouldn’t either and she had darker skin than me”* (maintenance). Similarly, *“I had skin cancer last year so I would, I absolutely must now [use sunscreen]”* (maintenance). Notably, some in the inaction stages indicated that a health scare might motivate them to change their attitude towards sunscreen. 

Unrelated health scares were also identified as stimulating a positive attitude towards sunscreen use. For instance, one interviewee who reported always using sunscreen stated, *“As you get a bit older you need to look after your health a bit more, there’s no excuses to say you didn’t know. I have had a recent health scare with my heart which has changed my outlook and made me make lifestyle changes”* (maintenance).

#### 3.1.2. Normative Beliefs 

Two themes were identified for subjective norms influencing sunscreen use—the influence of colleagues and the beliefs about the employer’s expectations. Irrespective of stage it was considered rare to see colleagues applying sunscreen in the workplace, *“I’ve never seen any of the boys with it”* (maintenance); *“I do, but I think there’s a lot that wouldn’t, I don’t see an awful lot of people putting it on to be honest”* (maintenance). There was an assumption that colleagues probably used sunscreen but applied it surreptitiously, *“I personally don’t see anyone putting it on, unless they put it on before they come to work, but I don’t actually ever see anyone applying it before going out on a walk. I’ve never seen a postman putting on sunscreen”* (maintenance). Some interviewees in the inaction stages acknowledged having observed colleagues apply sunscreen but felt that they did not need to use it due to having a lower risk skin type, *“The boys in there, their vans are full of it. Especially the lighter skinned boys, they will always have it with them”* (precontemplation). In the same way, *“I know two or three do put it on when they’re out you know, those that burn”* (precontemplation). 

Some participants observed that it was typically younger colleagues that used sunscreen: *“I would say I’m aware of it, because I see others put theirs on, particularly the younger ones”* (precontemplation). In line with this, some younger participants observed that older workers were less likely to use sunscreen: *“There is a guy here who will wind me up [tease, make fun of me] about putting sunscreen on. He is an older guy”* (maintenance). 

The employer was identified as an advocate for sunscreen yet disinclined to enforce its application, preferring to leave utilization decisions to individuals, *“We get told what they sort of recommend because of the sun on hot days but again it’s down to each individual if they choose not to do anything”* (maintenance). Across the stages of change, participants held the view that RMG would not enforce sunscreen application, *“We get the team briefings and they will say to you, ‘put on sunscreen’, but they don’t force you, it’s a personal preference”* (maintenance). Several participants expressed views on the notion of RMG providing sunscreen. The majority across the stages reported being in favor of this, noting that, if provided, they would in all likelihood use it as there was some indication its use was encouraged, *“I think the boss saying ‘there’s the sun cream, feel free to apply it’, people would go and apply it. If it wasn’t mentioned people wouldn’t always necessarily think about doing that”* (maintenance). Similarly, *“I think if it were [provided] in work and you come to work and it was red hot, I think folk would put it on”* (contemplation). 

The possibility of sunscreen use being enforced was roundly thought highly unlikely: *“[if compulsory] you’d have to do it then wouldn’t you, but I can’t ever see it being compulsory*” (precontemplation). Several participants thought it unlikely that RMG would provide sunscreen because of the allergy risk, *“They won’t provide it because you could be allergic to different types of sun cream, so you have to provide your own”* (maintenance), while others noted that they would prefer to have choice in product selection, *“I think if I was going to put sunscreen on my body I’d choose it myself”* (maintenance). 

#### 3.1.3. Control Beliefs 

Ability to control the application of sunscreen was identified as an important influence on its use. Those in the inaction stages perceived sunscreen to be difficult to use owing to the inconvenience of carrying a bottle when on delivery rounds, *“With everything I’m carrying I just think it’s too much to be carrying around all of the time”* (contemplation). In contrast, those in the action stages reported no such difficulties, *“I just have it in my kit bag. It is always there and I slap it on, factor 50”* (maintenance). Similarly, *“There aren’t really reasons you couldn’t do it. You should have it in the van”* (maintenance). 

Some in the inaction stages felt that sunscreen application was difficult, while those in the action stages indicated no such difficulties, tending to apply it prior to leaving home, *“It’s easy, I just stick it on in the morning before I come out”* (action). Reapplication was viewed as more difficult across the stages owing to sweat, *“As for reapplying, when you’ve got a film of sweat it’s very difficult to slap all that on top, and the odds of it drying would be slim as well”* (maintenance) and the likelihood of forgetting to take sunscreen out on a delivery round, *“I would reapply it unless I forgot to bring it on my round”* (maintenance). Those in the action stages whose delivery route involved use of a van reported that reapplication posed no difficulties, *“It’s easy, when you take your next bag out of the back of the van you put some on”* (maintenance). 

Control beliefs were linked to perceived availability, with some indicating that, if sunscreen were on hand, they would apply it, *“If someone handed it to me I might [use it] but I would never, I mean, I don’t think I have ever bought a bottle, I just use others’”* (precontemplation), while some noted that control was linked to remembering to bring sunscreen to work, *“There’s times when you leave the house and forget it”* (maintenance). 

Interestingly, when reflecting on their failure to use sunscreen at work, many interviewees in the inaction stages observed that they would use it when holidaying abroad and were fastidious when applying it to their children, *“I have children and every time it’s sunny they get doused in sun cream, so I suppose I should start doing it myself really…(laughs)”* (contemplation). 

### 3.2. Wide-Brimmed Hat

The stage of change distribution for wearing a wide brimmed hat when working outdoors in the summer contrasted sharply with that for sunscreen, with all but one participant being in the inaction stages (Table 3). The results that follow therefore focus on the early inaction stages of change and primarily those who have not considered adopting the sun safety practice. 

#### 3.2.1. Behavioral Beliefs 

Attitudes towards wearing a wide brimmed hat were decidedly negative, with views primarily driven by concerns about appearance, *“Those are ridiculous. They look stupid. I wouldn’t go out with that on”* (precontemplation); *“They are silly looking. I wouldn’t even think of wearing it”* (precontemplation). Interviewees acknowledged awareness of potential benefits afforded by a wide brimmed hat while expressing resistance to the idea of wearing one, *“I would definitely not [do that]. It looks stupid. I could see the benefits of it, but no, I wouldn’t do that. I don’t think the best design team in the world could come up with one that would look good”* (precontemplation); *“I wouldn’t wear one. That would just be fashion sense to me. It’s probably the right thing to do but I wouldn’t wear that”* (precontemplation); *“I’m sure it would benefit me, maybe your neck and stuff, stop it getting burnt, but I wouldn’t wear one”* (precontemplation). 

Some believed that a wide brimmed hat would make them the butt of jokes, “*It doesn’t look good. And you’ll find, being in … [location] … people here have a particular sense of humor and some of the customers will make fun of you*” (contemplation); “*When we are walking around the student areas, if we were walking around the streets with one of those wide brimmed hats on we would get slated*” (precontemplation). While concern regarding reactions from the public centered primarily on being the target of jokes, some felt that wearing a wide brimmed hat might make them the recipient of abuse, “*In the area where I deliver it’s like painting a target on your back, it really is*” (precontemplation); “*On the walks you can get abuse from some people and wearing that hat might get you some comments*” (contemplation).

Many in the inaction stages indicated that their negative attitude was determined by the UK climate and might be different if they lived elsewhere. In this regard, numerous participants referred to a proactive Australian sun safety culture and contrasted this to the UK, *“We don’t really have the climate for it; somewhere like Australia or somewhere that gets strong sun, they have respect for the sun and they’re told from a young age ‘go out with a hat, with sunscreen, with a long-sleeved shirt to keep the sun off’. Here we just go ‘it’s wet, it’s cold, where’s my hat?’”* (precontemplation). Australian sun safety culture was deemed responsible for making wide brimmed hats acceptable in that context, while remaining firmly unfashionable in the UK, *“There is nothing wrong with the design from a safety perspective, it is just how it looks. I think in somewhere like Australia no one would raise an eyebrow, but here I think it is off putting. Our society isn’t geared up for that sort of thing”* (contemplation). Similarly, *“Australian postmen, you could sell a load to them. They’re more used to the sun so they would be more used to wearing hats like that”* (precontemplation). 

In relation to both appearance and climate, some participants noted that a baseball-style cap offered a compromise in the sense of affording some coverage to the face and the top of the head while permitting a degree of sun exposure on rare sunny days, *“It is just trying to enjoy a bit of sun but at the same time being protected as well”* (precontemplation) and remaining fashionable, *“It just doesn’t look as nice as your cap, it really just isn’t a fashion statement”* (precontemplation). The single interviewee who identified as being in an action stage for this measure noted that he wore a wide-brimmed hat because he was bald and therefore required head coverage, and a baseball cap failed to provide neck coverage. This participant chose to wear his own wide-brimmed hat rather than that issued by RMG.

Several participants indicated that their attitude might change in response to sunburn, *“If I got really badly burned, yeah, that would make a difference and I would definitely start wearing it”* (contemplation) or skin cancer diagnoses, *“A friend of mine was told that he had skin cancer a little while ago. Now it’s cleared up, but if you’d something like that you would definitely consider [wearing a wide brimmed hat]”* (precontemplation). Furthermore, some indicated that attitude change might be possible if RMG was to introduce an attractive wide-brimmed hat, *“If Royal Mail can produce something that is going to be better I think that the guys would maybe use it”* (contemplation). 

#### 3.2.2. Normative Beliefs 

There was a widespread view that RMG advocated the use of a wide-brimmed hat yet did so with a “light touch”, providing hats while leaving individuals to decide whether to put them into use, *“They just provide it then it’s up to you to decide what to wear”* (precontemplation). Many interviewees noted that RMG did not enforce its use, *“They [RMG] would probably tell us we should be doing that but I think it’s one of those things that would slide. It’s not like they’re going to get on you about not wearing a wide brimmed hat”* (precontemplation); *“I know that RMG actually provides one of those so maybe they do expect us to wear them. It’s not really pushed onto you to wear it though. It is just available”* (precontemplation). Although some interviewees suggested that they would be unwilling to wear a wide brimmed hat even if it were company policy to do so, the contrary view was also expressed, *“If they made you wear one I suppose you’d do it wouldn’t you”* (precontemplation), and there was also a view that if enforced, over time it would become a cultural norm, *“If everybody started wearing them everybody would probably get over the embarrassment”* (contemplation).

#### 3.2.3. Control Beliefs

Across the stages, participants expressed the view that the wearing of a wide brimmed hat was within their control, *“I could do it if I wanted to, just, nah”* (precontemplation). Some in inaction stages noted that the wearing of a wide brimmed hat was difficult due to impracticalities such as catching on the strap of the mailbag when lifted over the head or blowing off in the wind. As one interviewee put it, *“We did get them a couple of years ago; I wore it for a day or two until I walked into a doorframe. It covers your eye, so practically, no I wouldn’t wear one now”* (precontemplation). Similarly, *“They’re just annoying. It could be red hot but if it’s windy they just go everywhere”* (precontemplation). Others noted that the hat tended to lose its rigidity when wet, making it impractical, *“I did have one once, and it got rained on and just collapsed”* (precontemplation); *“They’re too floppy, as soon as you wash it or it gets wet it never recovers its shape”* (precontemplation).

Several participants preferred to wear an RMG-issued baseball-style cap and take additional measures to cover the neck. For instance, *“The hat that Royal Mail issued a couple of years ago was too awkward and not particularly comfortable, actually pretty uncomfortable. The baseball cap seems to be better for me. What I do to make up for the lack of coverage on the neck is to put sunblock on and I find that works 100%”* (contemplation). Others used a baseball-style cap to protect the face while relying on shade provided by the upturned collar of a shirt to protect the neck, *“It’s a good idea to keep the sun off the back of your neck; with a polo shirt you can put your collar up so it isn’t too much of a problem really”* (precontemplation); *“Just a peak cap would do me because your collar is high enough on your polo, so you can just flick it up if you need to”* (precontemplation).

Some interviewees noted that wide brimmed hats were uncomfortable during hot weather, *“[We] are out getting really hot in this weather, walking four or five hours, hats are really hard to wear”* (precontemplation). Female participants noted that wide brimmed hats can be impractical on hot days for those with long hair: *“When it’s warm we girls would tend to wear our hair up and with that floppy hat it’s just not practical, it gets in the way. When you have your hair down it’s so warm you just want it up and out of the way. So that’s why [I don’t wear one], it’s not practical”* (precontemplation). Some in the inaction stages indicated that if RMG introduced a more comfortable and practical wide brimmed hat, they might be more inclined to use this measure. These interviewees also indicated that they would be more likely to use the hat if it were administered to all employees by default, *“If somebody got me one I would probably think about it”* (contemplation).

## 4. Discussion

### 4.1. Summary of Findings

This study adopted integrated theoretical perspectives to classify and explain UK postal delivery workers’ sun safety practices. Application of the TTM facilitated identification of current practices in accordance with the stages of behavior change. For sunscreen, approximately two thirds of interviewees reported being in the action stages, indicating that they typically applied this sun safety measure when working outdoors in the summer months. In contrast, almost all interviewees were in the inaction stages for the wearing of a wide-brimmed hat, with the vast majority of these indicating that they had not contemplated the possibility of applying this sun safety measure.

The TPB proved effective in providing a framework to account for factors informing sun safety practices between the inaction and the action stages of change. For sunscreen, those in the early inaction stages (precontemplation and contemplation) reported a negative attitude informed by a perception of low skin cancer risk arising from the UK climate and skin type; positive attitudes among those in the action stages (primarily the maintenance stage) were underpinned by awareness of skin cancer risk presented by the UK climate and skin type as well as by personal or family and friends’ experiences of skin cancer. Normative beliefs concerning colleagues’ views and the employer’s expectations about sunscreen application were consistent across the stages of change. There was a consistent perception that colleagues would not seek to influence others in regard to the regular use of sunscreen and would typically not apply sunscreen in view of one another. The employer was viewed as an advocate for sunscreen yet disinclined to enforce its application, preferring to leave utilization decisions to individuals. Control beliefs were stratified by inaction/action stage of change—those in the earlier stages were more likely to view sunscreen storage, application, and reapplication as inconvenient and difficult compared to those in the later stages. Stage of change stratification of beliefs was not evident for the wearing of a wide-brimmed hat because all but one interviewee expressed a negative attitude towards this sun safety measure. Participants’ views were driven by concerns about visual appearance and a perception that a wide-brimmed hat represents an excessive precaution in the UK climate. There was a normative belief that the employer would advocate wide-brimmed hats yet not enforce use. The wearing of a wide-brimmed hat was generally viewed as falling within an individual’s scope of control, though concerns about impracticality and discomfort were expressed.

### 4.2. Stage-Matched Interventions and the Theory of Planned Behavior 

This formative study contributes to the emerging literature on the application of psychological models of behavior change to classify and understand outdoor workers’ sun safety practices. The TTM stages of change framework proved effective for describing the extent to which postal delivery workers’ utilized sun safety practices, suggesting that it offers an effective basis for the evaluation of behavior change outcomes resulting from exposure to educational interventions in this sector, as has been demonstrated in the UK construction industry [22,26].

The stage of change perspective highlighted that educational interventions should take account of stage variance in practice within the target population. For instance, for sunscreen we found that interviewees were distributed across the inaction and the action stages of change, suggesting that interventions should contain heterogeneous stage-matched components. For sunscreen this could also include specific targeting within the inaction stages so that the intervention is tailored according to whether a participant is in the precontemplation or the contemplation stage (in the case of the former the intervention would not necessarily lead to behavior change to be successful). In other words, some components should be designed to encourage those in the inaction stages to move closer or into the action stages and others designed to help those in the latter stages to continue to remain there. In contrast, the very low level of wide-brimmed hat usage in our sample suggests that interventions concerning this measure ought to primarily focus on moving workers further through the inaction stages and then into action stages. Interventions to keep individuals in the action stages would only need to be considered later on. 

Ajzen [34] proposes that formative research to understand the factors underlying current behavior should be conducted as a precursor to the development of behavior change interventions. These interventions then target specific views across the TPB principle components, namely, *“(i) behavioral beliefs about the perceived consequences about performing the behavior, (ii) normative beliefs about the views of others, and (iii) control beliefs about the power of factors to facilitate or inhibit performance of the behavior”* (p. 757) [35]. In this study, we conducted what Epton [35] refers to as a “reasons elicitation phase” of formative research to identify TPB beliefs to target in subsequent interventions. We found that the TPB framework provided a good account of stage-specific factors underpinning postal delivery workers’ sun safety practices, with our results indicating that this theoretical perspective may usefully inform the content of stage-matched occupational sun safety interventions for this population. In this way, our study contributes to the growing body of literature demonstrating the efficacy of the TPB for understanding sun safety practices in diverse populations [18,19,20,21,22]. 

In demonstrating the presence of stage-specific beliefs underpinning occupational sun safety practices, our findings suggest that interventions that take account of stage-specific beliefs may stimulate behavior change beyond that achievable through generic interventions. Such has been demonstrated in the context of sun safety interventions involving university students [31], with similar results observed among workforce samples for occupational health challenges such as musculoskeletal disorders [23]. These findings point to the potential efficacy of stage-matched sun safety interventions for postal delivery workers that are stratified according to stage of change. Information about the health risk associated with current behavior can be directed at individuals in the pre-contemplation stage, while guidance on how to enact change is directed at those in the contemplation/preparation stages, and information on how to maintain change is provided to those in the action/maintenance stages. Stage-specific messages should be informed by the TPB behavioral beliefs, normative beliefs, and control beliefs that dominate at each stage. For instance, in our sample, there was a roughly equal split between inaction and action in relation to sunscreen use, with a negative attitude in the former group underpinned by a perception of low skin cancer risk arising from the UK climate and personal skin type. Personal experience of sunburn or skin cancer as well as that of close family or friends influenced the positive attitude towards sunscreen reported by those in the action stages. Normative beliefs about the views of colleagues indicated a perception that colleagues’ would not express a positive or negative opinion about others’ application of sunscreen, yet application tended to be done somewhat surreptitiously beyond the view of colleagues. Beliefs about the ability to control use of sunscreen were similarly split with those in the inaction stages more likely than those in the action stages to note storage, application, and reapplication difficulties. These findings suggest that educational interventions should address risk in relation to skin type, draw on the personal testimonies of postal delivery workers that have experienced skin cancer, highlight strategies for ease of application and reapplication, and encourage a culture of openness about applying sunscreen in the workplace. The emphasis on each of these elements should be contingent upon the stage of change targeted. 

Similarly, in relation to wide-brimmed hats, our study found a widespread negative attitude that was primarily driven by fashion concerns and a perception of the skin protection afforded being excessive for the UK climate. Impracticality associated with obstruction of vision was also raised as a factor discouraging use. Nevertheless, there was recognition of the sun protection benefits afforded by such hats, particularly in relation to neck coverage and a suggestion among contemplators that, if such a hat were provided to employees by default, usage may increase and potentially become a cultural norm. These findings suggest that educational interventions should address misconceptions about the likelihood of sunburn when working outdoors in the UK and highlight the degree of risk for areas of exposed skin that cannot be easily seen or covered with a shirt collar, such as the back of ears and the neck.

### 4.3. Practice Implications

Knowledge on factors underpinning positive and negative current practices may usefully inform the design of bespoke educational interventions targeted at specific sub-groups within the population. For instance, our findings show that the factors underpinning current positive practice tend to be distinct from those underpinning negative practice. Therefore, the content of interventions targeted at maintaining good practice might be different from those focused on encouraging a shift from inaction to action. Training providers should take into account the unique characteristics of the local postal delivery worker population and distinctions between sub-groups of the population defined in terms of whether or not current a practice is positive or negative in order to provide sun safety training that is specific to the needs of this population and stage-matched. 

We found a widespread negative attitude towards a wide-brimmed hat that was driven by concerns about appearance and impracticality. Farmers in the Midwestern United States similarly identified inconvenience as a disincentive to wearing a wide-brimmed hat, with 45% of survey respondents reporting such a hat gets in the way of their work [36]. A review of the literature on barriers to the wearing a wide-brimmed hat when working in the sun found that baseball-style caps were preferred by the majority of outdoor workers and these served to keep the sun out of the eyes, leaving little impetus for change [37]. These results highlight an imperative for the development of a hat suitable for company-wide distribution that provides shade to the face, the ears, and the neck without obstructing vision and remaining fashionable. An early study of US farmers found a preference for a cap with a flap at the back to cover the neck over a wide-brimmed hat [38]; such a strategy could be trialed among postal delivery workers. RMG might also encourage use by providing such a hat to all employees by default rather than requiring individuals to order a hat through the uniform website.

These findings also highlight scope for developments in the way that sunscreen is made available with a view towards altering perceptions concerning ease of use. Our findings are consistent with those of studies showing that outdoor workers perceive a host of issues, including inconvenience, as barriers to occupational sunscreen application [37]. The availability of pocket-sized single-application sachets might encourage reapplication during delivery rounds. In addition, text message sunscreen reminders have proven effective with other populations [39], including UK construction workers [29], and might be considered in the postal delivery context. 

Our findings further suggest that colleagues and the employer could be powerful normative influences on sun safety practice. This is in line with German research showing that outdoor workers who perceive high levels of workplace support for sun safety protect themselves from the sun during working hours, especially in terms of sunscreen, to a greater degree than those that perceive low levels of support [40]. One possible strategy then might be the adoption of company-wide cyclical sun safety promotion and intervention activities that contribute to the gradual normative shift. In this approach, the employer and the colleagues are perceived as expecting all to be proactive in the adoption of sun safety behaviors, gradually fostering proactive sun safety cultural norms.

### 4.4. Research Implications 

Sun safety interventions tailored to the characteristics and the needs of specific occupational groups have been shown to be effective [26,41]. As discussed above, there exists an imperative for the development and the implementation of educational interventions for UK postal delivery workers that are informed by the findings of formative studies such as those described herein, which provide guidance on targeting and content. Moreover, it is important that the efficacy of such interventions is evaluated both in terms of processes and outcomes so as to permit ongoing targeting at particularly at-risk individuals and refinement of SoC- and TPB-specific strategies. 

Our study focused on two sun safety behaviors that were selected because they fall within workers’ sphere of control and are potentially modifiable via educational interventions [13]. These measures represent just two among a wide range of practices within a comprehensive package of preventative and precautionary actions that exist within the control of the individual; measures such as seeking shade during deliveries, taking breaks in the shade, sunglasses, water consumption, long-sleeved shirts and trousers, and regular skin checks may also be utilized to varying degrees by postal delivery workers and accompanied by stage-specific influences. Future research might usefully explore the extent to which these and other sun safety behaviors find expression in the UK postal delivery workforce and the underpinning beliefs that inform behavioral decisions. The findings of such research could usefully inform the content of educational interventions that are theoretically and empirically underpinned and designed to encourage use of a wide range of sun safety measures.

### 4.5. Limitations 

This small-scale formative study did not seek to establish generalizable prevalence data on sun safety practices; as such, results concerning stage of change should be interpreted with caution. Nevertheless, our findings on the uptake of sunscreen when working outdoors during summer months are in line with the rate observed in earlier nationally representative research involving UK postal delivery workers [6]. This is the first study to examine use of a wide-brimmed hat within this population, and further work is required to establish whether the pattern of low usage identified herein is reflective of the sector more broadly. 

Participants self-selected into our study, raising the possibility that postal delivery workers with a particular interest in occupational sun safety or awareness of the risks associated with solar UV radiation exposure were more likely to participate. However, congruence between this study and earlier UK postal delivery worker research [6] in relation to sunscreen utilization rates suggests that such bias was unlikely. Adherence to sun safety practices was self-reported and potentially receptive to recall bias or bias towards socially desirable responses. In order to mitigate this possibility, we conducted interviews during the summer months at which time sun safety practices would be most relevant, thereby reducing the requirement to recall a previous summer season. In addition, previous studies of postal delivery workers have shown that subjective and objective measures of sun safety behavior are comparable [11]. Moreover, the high proportion of participants that reported being in the inaction stages of change suggests that socially desirable responding did not present a problem. The results are based on data drawn from a predominantly male sample and few participants indicated they were of skin type 5 or 6. The sample was broadly representative of the workforce in the regions involved and could be extended to examine whether similar findings emerge in other regions with a higher proportion of female postal workers and workers of skin types 5 and 6. The impact of employees’ socioeconomic status on the use of sunscreen may also merit further investigation as it may be linked to their decision to purchase this item. 

## 5. Conclusions

This study identified a host of theoretically grounded factors that influence UK postal delivery workers’ sun safety practices, indicating that the three principal components of the TPB offer a good account of stage-specific sun safety behavior. The pattern of sun safety practices and attendant beliefs identified in this formative research can be used to inform persuasive educational stage-matched messages tailored to the UK postal delivery workforce.

## Figures and Tables

**Table 1:** Participants’ skin type.

Skin Type	% Participants
Type 1: Pale skin, burns easily and rarely tans. Generally have light coloured or red hair and freckles.	11
Type 2: Fair skin that usually burns, but may gradually tan.	32
Type 3: Skin that burns with long or intense exposure to the sun but generally tans quite easily.	32
Type 4: Olive-coloured skin that tans easily, but could possibly burn with lengthy exposures to intense sunshine.	20
Type 5: Naturally brown skin, with brown eyes and dark hair. Skin darkens easily with sun exposure and only burns with excessive exposure to the sun.	5
Type 6: Black skin with dark brown eyes and black hair. Skin very easily darkens on exposure to sun and would very rarely, if ever, burn.	0

**Table 2:** Percentage of participants reporting each stage of change (sunscreen).

Stage of Change	% Participants
Precontemplation	18
Contemplation	18
Preparation	0
Action	11
Maintenance	53
Relapse	0

**Table 3:** Percentage of participants reporting each stage of change (wide-brimmed hat).

Stage of Change	% Participants
Precontemplation	82
Contemplation	15
Preparation	0
Action	0
Maintenance	3
Relapse	0
